# Editorial: Longevity with functionality and quality of life

**DOI:** 10.3389/fragi.2023.1281737

**Published:** 2023-11-15

**Authors:** Luiz Roberto Ramos, Eleonora d’Orsi, Eduardo Jardim Simões

**Affiliations:** ^1^ Department of Preventive Medicine, Federal University of Sao Paulo, Sao Paulo, Brazil; ^2^ Department of Public Health, Federal University of Santa Catarina, Santa Catarina, Brazil; ^3^ Department of Biomedical Informatics, Biostatistics and Medical Epidemiology, University of Missouri, Columbia, MO, United States

**Keywords:** factors associated with longevity, functional capacity in old age, quality of life in old age, elderly habits, risk factors for loosing function

The world is facing a longevity revolution, with an ever-growing aged population, with important socioeconomic and health implications. A large proportion of this elderly population has a chronic health condition without a cure, which may lead to disability. The concept of successful aging is associated with being able to live an independent and autonomous life, not necessarily free of any disease. Public health in this paradigm should look at health promoting interventions that prevent functional loss both in physical and mental terms thus promoting quality of life. The objective of this Research Topic was to gather evidence on risk factors for loss of functional capacity and effective interventions to preserve or improve functional capacity of the elderly on a population basis.

Few studies have addressed the potential of self-care behaviors among older adults to prolong independence in later life. Mielenz et al. used data from the National Health and Aging Trends Study cohort to evaluate the relationships of self-care behaviors with risk of impaired mobility and performance in activities of daily living. Eight baseline self-care behaviors were summarized using latent class analysis. Separately, longitudinal latent classes of mobility and ADLs were created. Two habitual baseline self-care behavioral patterns (46% favorable; 54% unfavorable) and three trajectories of change in mobility and ADLs disability (maintaining independence; shifting to accommodation/difficulty; shifting to assistance) emerged over time. Participants with a favorable baseline pattern had reduced risk in shifting to assistance class for mobility disability and for ADLs disability, and reduced risk for shifting to accommodation/difficulty class. Those with an unfavorable pattern greater risk of mortality compared to those with a favorable pattern. Interventions should encourage self-care behaviors constituting a favorable pattern.

Health behaviors and practices are determined by people’s perception, knowledge, and attitude about diet, physical activity, social networks, and healthcare. Bonfoh et al. used qualitative research to explore these factors among elderly in Japan and Cote D’Ivoire. Though elders in Japan lived longer than those in Cote D’Ivoir, both groups had strong social networks. However, Japanese depended on pension and insurance for income and healthcare, while Ivorians depended on their children and social network. Japanese resilience to aging was rooted on their fear of becoming a burden to their children and community, while Ivorian elders were considered socially and culturally useful to society. Knowledge about adequate diet and physical activity was much higher among elderly Japanese than Ivorian. Adequate diet and physical activity were more frequent among elderly Japanese than Ivorians. Healthy aging varied according to social systems, education, and knowledge about food and, in both contexts, physical activity, protein-energy balance in the diet and social networks are fundamental for healthy aging.

This study by Siqueira Junior et al. aims to analyze the association between walkability index and depressive symptoms and cognitive impairment and test the mediating role of moderate-vigorous physical activity in this relationship among older adults from Florianopolis, Brazil. Older adults residing in places with the highest walkability index were less likely to have a cognitive impairment, with a tendency for this relationship to be partially explained by the greater engagement in physical activities in places with greater walkability. This study brings insights into the importance of considering factors of the physical environment for policy planning to prevent and reduce the risks of cognitive impairment in older adults.

The high burden of COVID-19 hospitalizations and deaths made the elderly a priority group for prophylaxis. With the advent of the two mRNA vaccines, learning about the vaccine-induced immunity profile of the elderly was key for preparing an effective vaccination strategy. Muller et al. evaluated age-related differences in the longevity and magnitude of the induction of antibody responses post booster-vaccinations in two distinct cohorts aged below 60 and over 80 years Anti-SARS-CoV-2 spike-specific IgG and neutralization capacity waned rapidly after the initial vaccination schedule, more so among those aged over 80 years. However, elderly individuals’ immune response, including neutralization of Omicron variants, significantly increased with vaccine boosters. Researchers concluded that age-related differences in the humoral immune response were balanced by an additional booster vaccination, an appropriate strategy for successful vaccinations.

This qualitative study by Liang et al. aimed to investigate the acceptability of homebred exercise- and Tai-chi snacking in British and Taiwanese older adults of high and low physical function. Both snacking regimes were found to be convenient and easy to implement. The findings indicate that making Tai-chi snacking easier to master initially, building in progression, and adding some upper body movements in the exercise snacking may further enhance acceptability. The authors provide valuable insights for designing interventions to improve physical activity levels in older adults.

In this cross-sectional study conducted with baseline data from the Brazilian Longitudinal Study of Aging (ELSI-Brazil), Souza da Rosa et al. found that the combination of symptoms of depression and multimorbidity may increase functional impairments in both basic and instrumental activities of daily living, impairing self-efficacy, independence, and autonomy. The authors highlight the importance of early detection of combined risk factors for health promotion and disease prevention.

Fonseca et al. analyzed the relationships of the functional profile of older people admitted to long-term care units in Portugal with education, sex, age, and emotional state of mind. Data from the National Network of Integrated Continuous Care of Portugal were analyzed. In the first 90 days of hospitalization, activities of daily living and cognitive states improved, while mobility and instrumental activities of daily living worsened. All domains declined after 450 days of hospitalization. Older women with low education, those over 85 years old, and those who suffered from anxiety were pre-dominantly placed in the greater dependence group of. The authors highlight the importance of evaluating the functional status of persons in long-term care and the influence education exert on the recovery and rehabilitation of dependence.

There are multiple assessment tools for evaluating locomotion, especially lower-limb function, but most measure one dimension of movement in isolation or are not time-efficient, which may limit their use in community or clinical settings. O'Brien et al. assessed the reliability and convergent validity of a new multimodal functional locomotion assessment (FLA) for older adults. FLA assesses five major functional movements, and the overall task is timed: rise from a chair, walking gait, stair ascending/descending, obstacle avoidance, and descending to a chair. FLA showed high correlation with timed up-and-go performance and 6-min walk test distance, while its inter-rater reliability was very high. Findings warrant further investigation into the predictive validity of FLA as an assessment of lower-limb physical function among older adults.

